# A life-course and time perspective on the construct validity of psychological distress in women and men. Measurement invariance of the K6 across gender

**DOI:** 10.1186/1471-2288-10-68

**Published:** 2010-07-21

**Authors:** Aline Drapeau, Dominic Beaulieu-Prévost, Alain Marchand, Richard Boyer, Michel Préville, Sylvia Kairouz

**Affiliations:** 1 Département de psychiatrie, Université de Montréal, Montréal, Canada; 2 Centre de recherche Fernand-Seguin, Hôpital Louis-H. Lafontaine, Montréal, Canada; 3 Département de sexologie, Université du Québec à Montréal, Montréal, Canada; 4 École de relations industrielles, Université de Montréal, Montréal, Canada; 5 Département des sciences de la santé communautaire, Université de Sherbrooke, Sherbrooke, Canada; 6 Department of sociology and anthropology, Concordia University, Montréal, Canada

## Abstract

**Background:**

Psychological distress is a widespread indicator of mental health and mental illness in research and clinical settings. A recurrent finding from epidemiological studies and population surveys is that women report a higher mean level and a higher prevalence of psychological distress than men. These differences may reflect, to some extent, cultural norms associated with the expression of distress in women and men. Assuming that these norms differ across age groups and that they evolve over time, one would expect gender differences in psychological distress to vary over the life-course and over time. The objective of this study was to investigate the construct validity of a psychological distress scale, the K6, across gender in different age groups and over a twelve-year period.

**Methods:**

This study is based on data from the Canadian National Population Health Survey (C-NPHS). Psychological distress was assessed with the K6, a scale developed by Kessler and his colleagues. Data were examined through multi-group confirmatory factor analyses. Increasing levels of measurement and structural invariance across gender were assessed cross-sectionally with data from cycle 1 (n = 13019) of the C-NPHS and longitudinally with cycles 1 (1994-1995), 4 (2000-2001) and 7 (2006-2007).

**Results:**

Higher levels of measurement and structural invariance across gender were reached only after the constraint of equivalence was relaxed for various parameters of a few items of the K6. Some items had a different pattern of gender non invariance across age groups and over the course of the study. Gender differences in the expression of psychological distress may vary over the lifespan and over a 12-year period without markedly affecting the construct validity of the K6.

**Conclusions:**

This study confirms the cross-gender construct validity of psychological distress as assessed with the K6 despite differences in the expression of some symptoms in women and in men over the life-course and over time. Findings suggest that the higher mean level of psychological distress observed in women reflects a true difference in distress and is unlikely to be gender-biased. Gender differences in psychological distress are an important public health and clinical issue and further researches are needed to decipher the factors underlying these differences.

## Background

Psychological distress is a widespread indicator of mental health and mental illness in research and clinical settings and in public health. It combines mostly depression and anxiety symptoms that are indicative of a more or less intense feeling of emotional ill-being. As such, it is a common feature of most psychiatric disorders [1]. A recurrent finding from epidemiological studies and population surveys carried out in various countries is that women report a higher mean level and a higher prevalence of psychological distress than men [2-8]. Three main hypotheses have been raised to explain these gender differences. The first hypothesis is that women are more vulnerable than men to depressive symptoms. A number of biological (e.g., estrogen and progesterone), social (e.g.., learned helplessness) and psychological (e.g., rumination over current or past problems) factors and their interactions (e.g., gene-environment [9]) have been investigated to test this hypothesis but no consensus has emerged [10,11]. Women seem more responsive to stressful events related to their social network [12] or their parental role [13] but, in similar role configurations, they tend to experience an equal level of distress when faced with the same type of stress [3,14]. The second hypothesis is that women are more exposed than men to the risk factors associated with psychological distress. This hypothesis is supported for marital stress [15,16], domestic stress [5] and parental stress [13,16] but conflicting results have been found for job-related and financial stress [5,12-15]. The third hypothesis is that gender differences in psychological distress are, in part, a socio-cultural artifact resulting from differences in the way women and men perceive and express their distress. Thus the content and wording of some items may be more in line with the way women experience their feeling of distress.

This hypothesis is plausible given that the individual and collective experience of disease is partly bounded by cultural norms regarding the perception, expression and interpretation of psychological symptoms [6,17,18] and that these norms are often gender-related [19-21]. For instance, some somatic symptoms, such as a change in appetite or in body weight have been shown to characterize distressed and depressed women more than men [19-21]. According to Romans et al. [20], these symptoms are in agreement with the cultural norm that make physical appearance more of a concern for women. This claim is supported by a study conducted by Santor and his colleagues [22]showing that the item "distortion of body image" of the Beck Depression Inventory is more likely to be endorsed by women than by men whatever their level of distress. Similarly, the symptom "crying spells", which appears in several scales of psychological distress, may be more frequently endorsed by women since crying is culturally more "acceptable" for women than for men in most societies [20,22,23]. Men and women gain a common set of cultural norms and values in their infancy through nurturing and refine them over their lifetime through the social roles and experiences that provides them with opportunities to experiment and adapt the attitudes and behavior that are expected of them in various contexts. Thus, the reporting of psychological distress in a research or clinical setting may reflect not only the true level of distress experienced by women and men but also the influence of gender-related cultural norms regarding the perception and expression of distress. Furthermore, assuming that these norms may applied differently to younger vs. older people and that they may evolve over time, one would expect that gender differences in psychological distress would vary over the life-course and over time.

Jorm et al. [6] have noted a significant gender and age interaction where the mean level of distress decreased in women as they get older whereas a plateau was observed in men between the age of 20 to 44 years followed by a decrease. Leach et al. [24] have investigated the construct validity of psychological distress assessed with the 18-items version of the General Health Questionnaire (GHQ-18) [25] across gender in three age groups (i.e., 20-24; 40-44; 60-64) and conclude that this scale was not gender-biased despite differences in the endorsement of some items by women and men. Findings from Leach et al. [24] suggest that the variation of gender differences in psychological distress reflect true differences in distress over the life-course.

However, this conclusion may be premature given the scarce but convincing evidence regarding the difference in the construct and criteria validity of psychological distress observed across gender and age groups in various studies. For instance, Cheung [26] has demonstrated that, although the three-factor model (i.e., anxiety and depression; social dysfunction; loss of confidence) GHQ-12 fits women and men equally well, the correlations between these factors are higher in men. Fleishman and Lawrence [27] have found that two items (i.e., feeling calm and loss of energy) of the mental health dimension of the MOS 36-item Short-Form Health Survey (MH-SF-36) [28] function differently for men and women with men overrating these symptoms given their actual mental health status. In addition, some non somatic symptoms have been noted to occur more frequently in women or in men. In individuals with major depression, guilt feelings may be more likely to be endorsed by men than by women [19] and agitation may be more frequently reported in elderly men than elderly women [29]. In a study carried out in the general population, Romans [20] found that women with a high level of depression symptoms were more likely than men in the same state of mind to report loss of interest and thoughts of death.

Gender differences in the criteria validity of some scales used to assess psychological distress have also been observed. For example, there is some evidence that the GHQ-12 tends to underestimate the prevalence of affective disorders in women and overestimates it in men [30] whereas the GHQ-30 items and the Hopkins Symptom Checklist-25 items (SCL-25) [31] seem to better predict depression in men than in women [32,33]. Baillie [34] has found a small gender difference in the ability of the K10 [1,35] to predict some psychiatric disorders but he claimed that this difference was unlikely to impact epidemiological research.

Few studies have been published regarding the variation of the construct validity and psychometric characteristics of psychological distress scales across age-groups. Findings from Martin's study [36] suggest that, in younger adults, the three factors of the GHQ-12 items refer to self-esteem, stress and successful coping instead of anxiety and depression, social dysfunction and loss of confidence as observed in studies based on a larger age range. There is also some evidence that this scale may be less specific and more sensitive in younger patients than in older patients [30]. Fleishman and Lawrence [27] have shown that two items (i.e., feeling calm and loss of energy) of the MH-SF-36 function differently in seniors who tend to overate these symptoms given their actual mental health status. Finally, Ostroff et al.[37] have demonstrated that the factorial structure of the Psychological Distress Index of the Mental Health Inventory (PD-MHI) [38] differs in adolescents compared to that observed in the whole population.

In short, there is some indication that the construct validity of psychological distress in the general population and in patients may vary across gender and age groups. For instance, some symptoms (e.g., change in appetite or in body weight; crying spells) agree more with the culture of women than of men; a few distress items are more frequently endorsed by men (e.g., loss of energy; guilt feelings; agitation) or by women (e.g., loss of interest; thoughts of death); and the factorial structure of some distress scales (e.g., GHQ-12; PD-MH) may vary across age groups. The growing use of psychological distress as an indicator of the mental health of the population in large scale surveys and in epidemiological studies and as an outcome measure in the evaluation of intervention warrants in-depth studies regarding gender differences in the mean level of psychological distress.

The objective of this study was to investigate the construct validity of the K6 across gender in different age groups and over time. Kessler and his colleagues [1] carried out extensive analyses based on Item Response Theory to develop the K10 and K6, a smaller version of the K10. These 10 items were selected from a pool of 135 items derived from the symptoms used in the diagnostics of major depression and generalized anxiety disorder and in the positive affect domain. The selected items showed consistent severity values across socio-demographic groups (i.e., gender; education; age); the correlations between severity parameters across these groups ranging from 0.98 to 0.99 for the K6. The K10 and K6 have been used in major surveys in several countries where it was found to be a good predictor of anxiety disorder, with the exception of agoraphobia [39], and mood disorder [1,11,34,40-44].

In this study, data were analyzed within the framework of measurement and structural invariance. This framework defines a series of parameters that are tested for equivalence across groups. Measurement invariance refers to the relationship between observed and latent variables whereas structural invariance pertains to characteristics of the latent variables. The assessment of the measurement and structural invariance across groups follows a hierarchical procedure that has been developed over the years by several experts [45-51]. This procedure rests on a series of nested models where an increasing number of measurement and structural parameters are constrained to be equal across groups; each additional constraint defines an increasing level of invariance. Four main levels of measurement invariance are distinguished. The first level, configural invariance [45,50], relates to the equivalence of the factorial structure across groups; the only constraint is that the same items load on the same factors in all groups and, in a longitudinal perspective, at all cycles of the study. Demonstration of the configural invariance of the K6 across gender would be an indication that women and men use the same conceptual framework in their appraisal of psychological distress. The second level of measurement invariance is known as metric [45,50] or weak [52] invariance and it pertains to the invariance of factor loadings across groups and, in a longitudinal perspective, over time. Metric invariance suggests that the items of a scale have the same meaning for the groups under study [[50], Cheung, 1999 #427]or, as formulated by Brown [49], that a one-unit change in an item score is associated with the same change in the factor score in these groups. The third level of measurement invariance is scalar [45] or strong [50,52] invariance. With continuous items, scalar invariance refers to the equivalence of items intercepts across groups (and over time, if applicable) whereas, with ordinal items, it pertains to the equivalence of items thresholds, which are analogous to item difficulty in Item Response Theory [53]. Scalar invariance implies that the scaling of latent variable is equivalent in the compared groups. The fourth level of measurement invariance entails the equivalence of the residual variances, for continuous items, and of scale factors, for ordinal items. Scale factors represent the variance of the continuous latent response variable underlying the items [54]. Invariance of the scale factors implies that the scale is equally reliable in the groups under study. Finally, structural invariance concerns the equivalence of the latent factors variances and of the latent factor means across groups.

The level of inter-group invariance reached by a scale is an important issue since it has some bearing on the statistical analyses that can be conducted to compare these groups with that scale or, more precisely, on the interpretation of results. Metric invariance of a scale across groups validates the inter-group comparison of measures of associations (e.g., correlations) between this scale and other variables and of difference scores (e.g., pre/post difference) whereas scalar invariance allows valid comparisons of their mean score on this scale. Invariance of the scale factors is a necessary condition for analyses explicitly taking into account measurement error (e.g. structural equation modeling) but not for analyses based on observed values (as opposed to latent values). Equivalence of the latent factors variances is required to insure that the associations (e.g., correlations and regression coefficients) between the latent scale and other variables are not affected by a different range restriction of the latent scale between groups [48].

## Methods

### Data

The C-NPHS is an ongoing population-based longitudinal survey conducted every two-year since 1994 by Statistics Canada to assess the health status, lifestyle, and health care practices of Canadians. Access to the survey data files was granted by the Social Science and Humanities Council of Canada (SSHC) and Statistics Canada based on the scientific value of the research protocol and on the curriculum vitae of the researchers involved in this study. The target population of the C-NPHS is made up of Canadians aged 12 years and more and living in private households. At Cycle 1, in 1994, respondents were selected using a multi-level stratified sampling strategy to identify 20 095 households from which one person was randomly selected; the response rate was 86%. Over the next 6 cycles (i.e., 12 years) of the survey, 51% of respondents were lost to follow-up. Gender and age did not affect loss to follow-up but respondents with a higher level of psychological distress at Cycle 1 were more likely to drop out of the survey. Data were collected by computer-assisted phone interview (CAPI) by trained interviewers. Additional information regarding the design of the C-NPHS have been published elsewhere [55,56]. This study is based on data from cycles 1, 4 and 7 of the C-NPHS carried out, respectively, in 1994-1995, 2000-2001 and 2006-2007 and it focuses on respondents aged 18 years old and over. These three cycles were selected to cover the whole range of the survey period without overtaxing the already complex longitudinal analyses. The sample size totals 13019 for cross-sectional analyses of data from Cycle 1 and 6336 for longitudinal analyses of data from Cycles 1, 4 and 7.

The K6 is a uni-dimensional scale made up of 6 items asking respondents how often during the past 30 days they felt: so sad that nothing could cheer them up (item A); nervous (item B); restless or fidgety (item C); hopeless (item D); worthless (item E); that everything was an effort (item F). Each item is scaled from 0 (none of the time) to 4 (all of the time). The total score of psychological distress is computed by summing up the six items scores and it ranges from 0 to 24.

### Statistical analyses

Data were examined through multi-group confirmatory factor analyses (CFA) executed with Mplus-Version 4. Given the highly skewed distribution of the K6 items and their ordinal nature, CFAs were carried out from matrices of polychoric correlations and a robust mean adjusted weighted least squares estimator (WLSMV) as recommended by Brown [49], Flora and Curran [57] and Muthén and Muthén [51]. Three strategies were applied to take into account the unequal probability of selection due to the complex sampling method used in the C-NPHS. First, data were weighted with sampling weights calculated by Statistic Canada to insure the estimation of non biased parameters. Second, the bootstrap procedure (500 replications) was used to estimate the confidence intervals of the proportion of respondents endorsing each item and of the mean level of psychological distress (Table 1). Third, the threshold of statistical significance was fixed at α = 0.01 (instead of the traditional α = 0.05) in the assessment of the adequacy of the models with the chi-square statistics and the chi-square difference tests.

**Table 1:** Percent of respondents reporting that they felt each symptom of the K6 "none of the time" and "some of the time" or more^a ^in the past 30 days

	Men (n = 5730)		Women (n = 7289)		Factor loadings	
	
	%	**CI**^**b**^	%	**CI**^**b**^	Men	Women
A - So sad nothing could cheer you up					.76	.77
None of the time	69	68-71	58	56-59		
Some of the time or more	9	8-10	16	15-17		
B - Nervous					.57	.58
None of the time	46	44-47	38	37-39		
Some of the time or more	21	20-22	27	27-29		
C - Restless or fidgety					.54	.60
None of the time	51	50-53	50	48-52		
Some of the time or more	23	22-24	23	22-25		
D - Hopeless					.92	.92
None of the time	87	86-88	81	80-82		
Some of the time or more	5	4-6	8	7-9		
E - Worthless					.86	.85
None of the time	93	92-93	89	88-89		
Some of the time or more	3	2-3	5	4-5		
F - Everything is an effort					.65	.68
None of the time	55	53-57	49	47-50		
Some of the time or more	19	19-22	23	22-25		
Mean level of psychological distress	3.04 2.93 to 3.14		3.70 3.58 to 3.81			

^a ^Due to the small percent of respondents in the categories "some of the time", "most of the time" and "all of the time", the percents for these categories are pooled together in the Table. For ease of reading, the percent of respondents reporting "a little of the time" is not shown in the Table; it corresponds to 100 minus the percents shown in the categories "none of the time" and "some of the time or more" for each item.

^b ^Confidence interval at the 0.95 level.

### Preliminary analyses

In preliminary analyses, the uni-dimensional structure of the K6 was tested separately for each subgroup (i.e., each gender, in the pooled sample, in each age group and at each cycle of the C-NPHS under study). The adequacy of these models was assessed with two relative fit indexes - the comparative fit index (CFI) and the Tucker-Lewis index (TLI) - and one absolute fit index - the root mean square error of approximation (RMSEA). These indices are based on the chi square measure and take the degrees of freedom into account; the RMSEA also controls for sample size. According to Hu and Bentler [58], values greater than .95 for the CFI and the TLI and smaller than .06 for the RMSEA indicate a good fit of the model.

An omnibus test of the equality of the matrices of polychoric correlations and of the threshold structure of the items across gender was also carried out, separately, for the pooled sample and for each age group. This test serves to verify whether there are gender differences in the structure of the data [48]. Analyses pertaining to the measurement and structural invariance of the K6 are undertaken if these matrices are found to diverge across gender.

### Measurement and structural invariance

Measurement and structural invariance across gender was assessed, first, cross-sectionally with data from cycle 1 of the C-NPHS for the pooled sample and, separately, for each age group (18-39; 40-64; 65+) and, second, longitudinally with cycles 1 (1994-1995), 4 (2000-2001) and 7 (2006-2007) of the survey. Longitudinal analyses were restricted to respondents with complete K6 data for cycles 1, 4 and 7; sampling weights for cycle 7 were used for these analyses.

The assessment of cross-sectional and longitudinal invariance across gender followed roughly the same procedure except that, for the latter, the dependence of repeated observations over time was taken into account. This was achieved by modeling the latent distress score at cycles 1, 4 and 7 as three separate variables nested within individuals and by allowing correlations between the three cycles of the survey for the latent factor score and for each item's residual [48,59,60]. These correlations may be understood as test-retest correlations or as indicators of the longitudinal stability of the latent construct and of the measurement error terms (i.e., the residuals) over a six-year (Cycles 1 and 4; Cycles 4 and 7) and a twelve-year period (Cycles 1 and 7).

The goodness of fit of the configural model was assessed with the CFI, the TLI and the RMSEA. Starting from metric invariance, the chi-square difference test (Δχ^2) ^was used to assess the goodness-of-fit of a model by comparing it to the model in which it is nested (i.e., the models with vs. without the additional constraint). Thus the metric invariance model is compared to the configural invariance model; the scalar invariance model is compared to the metric invariance model, and so on. A non significant Δχ^2 ^(in this study, p > .01) indicates that the more constrained model does not worsen the fit to the data and may be accepted. Given a significant difference between these models, the more constrained model is rejected and the investigation of measurement invariance does not proceed further. Tau equivalence of the items was also investigated. Tau equivalence is a stricter form of metric invariance in which the factor loadings are also constrained to be equal between all items [49]. The adequacy of this model is tested by comparison with the metric invariance model obtained. Failing to reach Tau equivalence would suggest that adding the individual score of the six items of the K6 may not provide an adequate scoring of psychological distress. Finally, in the investigation of the last level of measurement invariance, the scale factors of a group are compared to the scale factors of the reference group for which it is fixed to one for each item. A scale demonstrates invariance of its scale factors if (a) it presents both metric and scalar invariance and (b) the scale factor for each latent item is equal to one in each group (and each cycle of the study in a longitudinal study). The adequacy of the model of equivalence of scale factors is tested against the model of scalar invariance.

Two level of structural invariance were investigated: invariance of the latent factors variances and invariance of the latent factor means. The former is tested against the final model of measurement invariance whereas the latter is compared to the model of latent factors variances. Longitudinal invariance is almost meaningless for these two levels of structural invariance because the distribution of the latent factor has to be standardized at each of the three cycles for the group of reference (here, men) to insure model identification. Thus, by definition, the distribution of the latent factor scores for men has a mean of zero and a variance of one at each of the three cycles. Consequently, longitudinal invariance of the factor variances strictly implies that the ratio between the latent factor variances of men and women stays the same for each cycle and longitudinal invariance of the factor means strictly implies that the difference between the latent factor means of men and women stays the same (in standardized units) for the three cycles.

### Longitudinal stability

As mentioned earlier, longitudinal models are defined by correlations between the three cycles of the survey for the latent factor score and for each item's residual to take into account the inter-dependence of the repeated measures. These correlations, occasionally called test-retest correlations [60], represent the relation between repeated measures of the same parameter over time (i.e. how much the score for a parameter can be predicted from its value in a previous cycle). As such, they may be considered as indicators of the stability of a score over time (i.e., of the longitudinal stability of the score [59]. A correlation close to 1 implies that the parameter can be precisely predicted from its previous value (i.e. it is highly stable over time like a personality trait and is not really affected by contextual elements). A correlation close to 0 implies that the parameter cannot be predicted from its previous value (i.e. it is less stable and potentially more context- or state-dependant).

Following Marsh and Grayson [59], the longitudinal stability of the latent factor and of the residual of each item was investigated after the assessment of equal scale factors (i.e. after measurement invariance was completely assessed). A scale demonstrates complete invariance of the longitudinal stability across gender if (a) it is metrically invariant and (b) the value of the longitudinal stability of every item's residual and of the latent factor is equivalent between groups [59,60]. In this study, the longitudinal stability of the construct was assessed over a six-year (Cycles 1 and 4; Cycles 4 and 7) and a twelve-year period (Cycles 1 and 7) for each item's residual and for the latent factor.

To assess the invariance of the longitudinal stability requires using a longitudinal multi-group CFA approach. It is a complex enterprise and it has rarely been undertaken. Thus, the implications of invariance at this level are not clearly defined in the literature. From a theoretical point of view, indicators of longitudinal stability describe the group's equivalences/differences of the temporal behavior of both the latent factor and each item's residual. For example, if the longitudinal stability of the latent factor is higher for women than for men, the capacity of the latent distress scale to predict an event at a subsequent cycle of the survey will be higher for women. Invariance of the longitudinal stability of the latent factor and of each item's residual is not required for most types of analyses but, in theory, it could be an important issue when the scale is used to predict future events since the predictive power of a scale depends both on the relationship between the scale and the predicted event and on the stability of the scale over time. This level of invariance may be especially crucial to insure the validity of longitudinal analyses with a lagged dependent variable because the coefficients associated with this type of variable represent the relationship between a dependent variable and its value at the previous wave, which is akin to longitudinal stability.

### Partial invariance

Except for configural invariance, partial invariance was explored, whenever complete invariance did not hold for a model, by relaxing the equivalence constraint for failing items (i.e., letting them free to vary across gender). Partial invariance implies that the parameter under study is invariant for some but not all items. It is an acceptable alternative when complete invariance cannot be reached [47]. The factor-ratio procedure developed by Cheung and Rensvold [47] was used to identify non invariant items at the metric and subsequent levels of invariance. An item that is shown to be non equivalent across groups at a specific level of invariance remains unconstrained in the investigation of the next levels of invariance.

## Results

At baseline, the sample totaled 7289 (56%) women and 5730 (44%) men; 44% (n = 5748) were young adults (18-39 years old), 36% (n = 4746) were middle-aged (40-64 years old) and 19% (n = 2525) were seniors (65 years old and over). The mean level of distress at cycle 1 was higher in women (3.70 CI 3.58 to 3.81) than in men (3.04 CI 2.93 to 3.14) (Table 1). The symptoms least frequently endorsed by both men and women were feeling "hopeless" (item D) and "worthless" (item E); 3% to 8% of respondents reported feeling these symptoms some of the time or more in the past 30 days. Those most frequently endorsed at that level of intensity were feeling "nervous", "restless or fidgety" and "everything is an effort" (Table 1). A higher percent of women than of men reported feeling "so sad nothing could cheer you up" (item A: 16% vs. 9%), "nervous" (item B: 27% vs. 21%), "hopeless" (item D: 8% vs. 5%) and "worthless" (item E: 5% vs. 3%) some of the time, most of the time or all of the time in the past 30 days.

### Preliminary analyses

The uni-dimensional structure of the K6 was confirmed in each subgroup (i.e., each gender, overall, in each age group and at each cycle of the C-NPHS) based on the three goodness of fit indexes (CFL, TLI, RMSEA) only after the correlation between the residuals of item B (nervous) and item C (restless or fidgety) was taken into account and specified in the models. This relationship makes sense since both items tap on the anxiety aspect of psychological distress. However, the correlations between these items, which ranged between r = .12 and r = .25, was not high enough in the pooled sample nor in any age group to establish a formal second dimension for the K6. The relationship between items B and C was specified in all subsequent models of measurement and structural invariance. The omnibus tests carried out for the pooled sample and in each age group were statistically significant (*p *< 0.01), thus indicating that some parameters of the K6 items are not equivalent across gender.

### Cross-sectional measurement and structural invariance across gender

The unconstrained factor loadings for each item were moderate to high in the pooled sample (range .54 to .92) (Table 1) and in the three age groups (range .47 to .92). The highest factor loadings were those associated with the items D (hopeless) and E (worthless) in both gender in all groups. Most gender differences in factor loadings were smaller than .03; the largest difference being that for the item C "restless or fidgety" in young adults (Men .49; Women .57). Tables 2, 3, 4 and 5 show the fit indexes of the successive models of invariance for the pooled sample and for each age group. The CFI, TLI and RMSEA of the configural model (M1) indicate that this model fits the data well in the pooled sample and three sub-samples. Complete metric invariance of the K6 across gender was reached since the metric model (M2) did not significantly worsen the fit to the data compared to the configural model. These results suggest that the concept of psychological distress (i.e., configural invariance), as assessed with the K6, and the factor loadings for each item (i.e., metric invariance) are similar in women and men overall and in each age group. This implies that associations (e.g., correlations) between the latent K6 and other variables and difference scores (e.g., pre/post difference) on the latent K6 can be validly compared across gender in all age groups under study. However, the hypothesis of the Tau equivalence of the items (M3) was rejected in the pooled sample and in all age groups. Thus summing up the items scores does not appear to be the optimal scoring system for the K6.

**Table 2:** Measurement and structural invariance across gender at cycle 1 Pooled sample (n = 13019)

Level of invariance	**X**^**2**^ df p value	CFI	TLI	RMSEA	**Δχ**^**2**^ **df**^**c**^ p value
Measurement invariance					
M1. Configural	123.7 14 < .0001	0.993	0.993	0.035	N/A
M2. Metric (vs. M1)	100.1 16 < .0001	0.995	0.996	0.028	13.3 5 0.0206
M3. Tau equivalence (vs. M2)	1783.0 20 < .0001	0.894	0.926	0.116	1495.8 5 < .0001
M4. Scalar - Complete (vs. M2)	111.28 24 < .0001	0.995	0.997	0.024	25.9 11 0.0066
M5. Scalar - Partial ^a ^(vs. M2)	98.7 22 < .0001	0.995	0.997	0.023	9.6 8 0.2969
M6. Scale factor - Partial^b ^(vs. M5)	91.8 23 < .0001	0.996	0.997	0.021	8.9 9 0.4478
Structural invariance					
M7. Latent variance (vs. M6)	59.9 17 < .0001	0.997	0.998	0.020	0.3 1 0.6083
M8. Latent means (vs. M7)	193.9 15 < .0001	0.989	0.99	0.043	83.2 2 < .0001

^a ^The constraint of equal item threshold was relaxed for item A (so sad nothing could cheer you up) and item C (restless or fidgety).

^b ^Complete invariance of the scale factors could not be investigated since items A and C had to be unconstrained in the preceding model (i.e., scalar invariance model). No additional items were freed to reach partial scale factor invariance.

^c ^The degrees of freedom for the chi-square tests are adjusted for the WLSMV estimator and do not correspond to difference of degrees of freedom between the more constrained and the less constrained model.

**Table 3:** Measurement and structural invariance across gender at cycle 1 Young adults (18-39 years old; n = 5748)

Level of invariance	**X**^**2**^ df p value	CFI	TLI	RMSEA	**Δχ**^**2**^ **df**^**c**^ p value
Measurement invariance					
M1. Configural	66.3 14 < .0001	0.993	0.993	0.036	N/A
M2. Metric (vs. M1)	54.7 15 < .0001	0.995	0.995	0.03	8.7 4 0.0679
M3. Tau equivalence (vs. M2)	1108.1 19 < .0001	0.855	0.893	0.141	972.7 5 < .0001
M4. Scalar - Complete (vs. M2)	84.0 25 < .0001	0.992	0.996	0.029	41.5 13 <.0001
M5. Scalar - Partial ^a ^(vs. M2)	60.3 21 < .0001	0.995	0.997	0.026	11.3 8 0.1828
M6. Scale factor - Partial^b ^(vs. M5)	62.1 23 < .0001	0.995	0.997	0.024	15.5 10 0.1142
Structural invariance					
M7. Latent variances (vs. M6)	48.1 17 < .0001	0.996	0.997	0.025	3.8 1 0.0522
M8. Latent means (vs. M7)	158.2 16 < .0001	0.981	0.983	0.056	69.7 2 < .0001

^a ^The constraint of equal item threshold was relaxed for item C (restless or fidgety) and item F (everything is an effort).

^b ^Complete invariance of the scale factors could not be investigated since items C and F had to be unconstrained in the preceding model (i.e., scalar invariance model). No additional items were freed to reach partial scale factor invariance.

^c ^The degrees of freedom for the chi-square tests are adjusted for the WLSMV estimator and do not correspond to difference of degrees of freedom between the more constrained and the less constrained model.

**Table 4:** Measurement and structural invariance across gender at cycle 1 Middle-aged adults (40-64 years old; n = 4746)

Level of invariance	**X**^**2**^ df p value	CFI	TLI	RMSEA	**Δχ**^**2**^ **df**^**a**^ p value
Measurement invariance					
M1. Configural	56.8 13 < .0001	0.993	0.993	0.038	N/A
M2. Metric (vs. M1)	47.1 15 < .0001	0.995	0.995	0.03	6.9 5 0.2282
M3. Tau equivalence (vs. M2)	579.1 19 < .0001	0.907	0.936	0.111	508.1 5 < .0001
M4. Scalar (vs. M2)	57.8 24 < .0001	0.994	0.997	0.024	21.5 13 0.0643
M6. Scale factor (vs. M4)	58.9 27 0.0004	0.995	0.997	0.022	21.9 15 0.1097
Structural invariance					
M7. Latent variance (vs. M6)	36.5 19 0.0090	0.997	0.998	0.02	0.009 1 0.9228
M8. Latent means (vs. M7)	90.4 16 < .0001	0.988	0.99	0.044	33.2 2 < .0001

^a ^The degrees of freedom for the chi-square tests are adjusted for the WLSMV estimator and do not correspond to difference of degrees of freedom between the more constrained and the less constrained model.

**Table 5:** Measurement and structural invariance across gender at cycle 1 Seniors (65 years old and over; n = 2525)

Level of invariance	**X**^**2**^ df p value	CFI	TLI	RMSEA	**Δχ**^**2**^ **df**^**c**^ p value
Measurement invariance					
M1. Configural	35.8 14 < .0001	0.994	0.994	0.035	N/A
M2. Metric (vs. M1)	22.9 15 < .0001	0.998	0.998	0.020	0.634 5 0.9864
M3. Tau equivalence (vs. M2)	249.1 19 < .0001	0.934	0.955	0.098	207.3 5 < .0001
M4. Scalar - Complete (vs. M2)	60.8 23 < .0001	0.989	0.994	0.036	54.6 12 < .0001
M5. Scalar - Partial ^a ^(vs. M2)	32.7 20 < .0001	0.996	0.998	0.022	20.9 9 0.0132
M6. Scale factor - Partial^b ^(vs. M5)	41.3 22 < .0001	0.994	0.997	0.026	22.9 10 0.0112
Structural invariance					
M7. Latent variance (vs. M6)	26.6 16 < .0001	0.997	0.998	0.023	0.324 1 0.5691
M8. Latent means (vs. M7)	88.4 15 < .0001	0.979	0.982	0.062	36.7 2 < .0001

^a ^The constraint of equal item threshold was relaxed for item C (restless or fidgety) and item D (hopeless).

^b ^Complete invariance of the scale factors could not be investigated since items C and D had to be unconstrained in the preceding models (i.e., metric or scalar invariance model). No additional items were freed to reach partial scale factor invariance.

^c ^The degrees of freedom for the chi-square tests are adjusted for the WLSMV estimator and do not correspond to difference of degrees of freedom between the more constrained and the less constrained model.

Compared to the metric model, the scalar model (M4) significantly worsened the fit to the data in the pooled sample (Δχ^2 ^= 50; p <.0001) and in the younger (Δχ^2 ^= 41.5; p = .0001) and older group (Δχ^2 ^= 54.6; p < .0001). Thus, complete scalar invariance was reached only in the middle-aged group. In the pooled sample and in the younger and older age groups, the constraint of equal thresholds across gender had to be relaxed for some items to attain partial scalar invariance (M5). The item thresholds for item C (restless or fidgety) was allowed to vary across gender in the pooled sample, in young adults and in seniors. This constraint was also relaxed for items A (so sad nothing could cheer you up), F (everything was an effort) and D (hopeless) in, respectively, the pooled sample (Table 2) and the younger (Table 3) and older (Table 5) age groups.

By definition, items with scalar non-invariance in a specific group cannot reach scale factor invariance in that group. However, no additional items had to be unconstrained to reach partial invariance of the scale factor (M6). Thus, both partial scalar and partial scale factor invariance were reached for the pooled sample, for young adults and for seniors, with four invariant items out of six in each case whereas complete scalar and scale factor invariance was demonstrated for middle-aged. Consequently, since partial invariance is an adequate alternative to complete invariance, the highest level of measurement invariance (except Tau equivalence) was reached for the pooled sample and the three age groups. This suggests that the mean and the variance of the latent K6 and the associations between the latent K6 and other variables can be validly compared between men and women aged 18 and over in cross-sectional studies.

Regarding structural invariance, complete invariance of the latent factor variances (M7) across gender was established for the pooled sample and in each age group thus indicating that the latent range used by women and men is equivalent in the age groups under study. However, the invariance of latent factor means (M8) across gender was not demonstrated. Since at least partial scalar invariance was reached in every sample, the lack of invariance of the latent factor means points to a genuine gender difference in the mean level of psychological distress. More precisely, the mean level of distress appears to be systematically higher in women than in men in the three age-groups under study. The parametric values of the final model of cross-sectional invariance for the pooled sample and for the three age groups are shown in Table 6.

**Table 6:** Parameters of the final cross-sectional measurement invariance model at cycle 1

	Pooled (n = 13019)	Young adults (n = 5748)	Middle-aged (n = 4746)	Seniors (n = 2525)
A - So sad nothing could cheer you up				
Factor loadings	.77	.79	.75	.81
Threshold 0-1	0.50/0.42	0.40	0.48	0.73
Threshold 1-2	1.32/1.24	1.29	1.25	1.44
Threshold 2-3	2.08/2.05	2.12	2.06	2.11
Threshold 3-4	2.78/2.63	2.78	2.64	2.70
B - Nervous				
Factor loadings	.58	.50	.63	.67
Threshold 0-1	-0.13	-0.34	-0.02	0.36
Threshold 1-2	0.79	0.72	0.82	1.07
Threshold 2-3	1.69	1.71	1.67	1.84
Threshold 3-4	2.24	2.31	2.17	2.36
C - Restless or fidgety				
Factor loadings	.58	.54	.58	.65
Threshold 0-1	0.03/0.17	-0.16/-0.01	0.21	0.36/0.60
Threshold 1-2	0.76/0.92	0.58/0.80	0.96	1.08/1.29
Threshold 2-3	1.61/1.71	1.48/1.59	1.79	1.81/2.04
Threshold 3-4	2.02/2.23	1.87/2.21	2.25	2.36/2.37
D - Hopeless				
Factor loadings	.92	.92	.91	.94
Threshold 0-1	1.13	1.08	1.19	1.23/1.47
Threshold 1-2	1.68	1.67	1.70	1.77/1.95
Threshold 2-3	2.36	2.38	2.38	2.52/2.48
Threshold 3-4	2.74	2.71	2.76	3.63/2.82
E - Worthless				
Factor loadings	.85	.86	.87	.81
Threshold 0-1	1.46	1.42	1.52	1.57
Threshold 1-2	1.94	1.99	1.92	2.10
Threshold 2-3	2.49	2.60	2.42	2.66
Threshold 3-4	2.78	2.93	2.67	2.86
F - Everything is an effort				
Factor loadings	.63	.64	.69	.68
Threshold 0-1	0.14	-0.03/0.08	0.24	0.37
Threshold 1-2	0.87	0.74/0.91	0.96	1.06
Threshold 2-3	1.54	1.39/1.65	1.64	1.73
Threshold 3-4	2.08	2.02/2.18	2.31	2.06

^a ^The threshold over the diagonal is for men; the parameter below the diagonal is for women. A single value indicates that the threshold is similar for men and women (i.e., gender invariance for the threshold).

### Longitudinal measurement invariance across gender

Table 7 shows the goodness-of-fit indices pertaining to the measurement and structural invariance of the K6 across gender over time and Table 8 presents the main parametric values of the final longitudinal model. The longitudinal measurement invariance may be appreciated from two intertwined points of view: first, the inter-group invariance (women vs. men) at cycles 1, 4 and 7 of the C-NPHS; and, second, the intra-group invariance (within women; within men) over time. Configural invariance (L1) was established thus suggesting that the conceptual framework used to assess psychological distress is similar across gender at cycles 1, 4 and 7 and over the twelve years of the study in both women and men. Complete metric invariance (L2) of the K6 could not be demonstrated. However, partial metric invariance (L3) was reached after relaxing the constraint of equal factor loadings for some items. Regarding inter-group invariance, this constraint was relaxed for item C (restless or fidgety) at cycle 1. As can be seen in Table 8, freeing the factor loadings of this item at cycle 1 had an impact on the longitudinal metric invariance of men (Factor loadings Cycle 1 = .48; Cycles 4 and 7 = .61) but not on that of women (Factor loadings Cycles 1, 4 and 7 = .61). In addition, regarding within-group invariance, the constraint of equal factor loadings over time was relaxed in men for items B (nervous) and F (everything was an effort) in cycle 1 (vs. cycles 4 and 7) but not in cycles 4 and 7; these factor loadings were invariant across gender (Table 8). In summary, metric invariance across gender was found for five items (A, B, D, E and F) at cycle 1 and all items at cycles 4 and 7 whereas metric invariance over time was partial for men (3 items invariant: A, D and E) and for women (4 items invariant: A, C, D and E). Thus, it would appear that the meaning of some items of the K6 has somewhat evolve over the course of the study although this evolution does not seem to affect the construct validity of the scale across gender. Consequently, findings on the longitudinal metric invariance suggest that associations (e.g., correlations) between the latent K6 and other variables and difference scores on the latent K6 can be validly compared across gender and over a 12 years period.

**Table 7:** Longitudinal measurement invariance across gender at cycles 1, 4 and 7 Pooled sample (n = 6336^a^)

Level of invariance	**X**^**2**^ df p value	CFI	TLI	RMSEA	**Δχ**^**2**^ **df**^**e**^ p value
Measurement invariance					
L1. Configural	240.3 97 < .0001	0.982	0.993	0.022	N/A
L2. Metric (vs. L1)	263.8 98 < .0001	0.98	0.991	0.023	71.0 21 <.0001
L3. Metric - Partial^b ^(vs. L1)	224.4 100 < .0001	0.985	0.994	0.02	25.5 19 0.1462
L4. Scalar - Partial^c ^(vs. L3)	309.9 118 < .0001	0.976	0.992	0.023	145.8 40 <.0001
L5. Scalar - Partial^d ^(vs. L3)	233.6 110 < .0001	0.985	0.994	0.019	45.2 27 0.0154
L6. Scale factor - Partial^c ^(vs. L5)	234.1 114 < .0001	0.985	0.995	0.018	48.0 32 0.0342
Structural invariance					
L7. Longitudinal stability (vs. L6)	111.6 60 < .0001	0.994	0.996	0.016	5.8 5 0.3299
L8. Latent variance (vs. L7)	97.5 54 < .0001	0.995	0.996	0.016	4.4 4 0.3522
L9. Latent means (vs. L8)	198.3 53 < .0001	0.982	0.986	0.029	114.7 6 <.0001
L10. Latent means - Longitudinal (vs. L8)	105.4 55 < .0001	0.994	0.995	0.017	15.0 6 0.0199

^a ^Number of respondents with complete data for cycles 1, 4 and 7.

^b ^The constraint of equal factor loadings was relaxed at cycle 1 for item C (restless or fidgety) and over time in men for items B (nervous), C (restless or fidgety) and F (everything was an effort) and for women for items B and F.

^c ^Complete invariance of the scale factors could not be investigated since some items had to be unconstrained in the preceding models.

^d ^The constraint of equal items thresholds was relaxed for items A (so sad nothing could cheer you up), B and D (hopeless) at cycle 4 and for items C (restless or fidgety), D and F at cycle 7. In men, item A was freed whereas, in women, the constraint of equal item thresholds was relaxed for all items except E.

^e ^The degrees of freedom for the chi-square tests are adjusted for the WLSMV estimator and do not correspond to difference of degrees of freedom between the more constrained and the less constrained model.

**Table 8:** Parameters^a ^of the final longitudinal model (n = 6336)

	**Factor loadings**^**b**^		**First threshold **^**b,c**^			**Longitudinal stability**^**d**^	
	Cycle 1	Cycles 4 and 7	Cycle 1	Cycle 4	Cycle 7	6 years	12 years
Item A So sad ...	0.78	0.78	0.50	M 0.82 W 0.77	0.82	0.05	0.04
Item B Nervous	0.56	0.67	-0.19	M 0.32 W 0.29	0.32	0.20	0.23
Item C Restless ...	M 0.48 W 0.61	0.61	M -0.00 W 0.16	0.42	M 0.42 W 0.47	0.20	0.15
Item D Hopeless	0.92	0.92	1.24	M 1.24 W 1.51	M 1.24 W 1.38	0.05	0.04
Item E Worthless	0.87	0.87	1.59	1.59	1.59	0.05	0.04
Item F ... effort	0.63	0.77	0.17	0.82	M 0.70 W 0.80	0.05	0.04

^a ^All the scale factors equal 1.00 in women and men; thus they are not presented in this Table.

^b ^A single value indicates that the parameter is invariant across gender. When a gender difference is observed, M identifies the value for men and W the value for Women.

^c ^Only data pertaining to the first threshold (i.e., between "none of the time" and "a little of the time") are presented.

^d ^Correlations between the residuals of the items over time.

The invariance of items threshold across gender and over time cannot be investigated for those items whose factor loadings were freed in the assessment of metric invariance; thus complete longitudinal scalar invariance could not be reached. Maximal scalar invariance (L4) across gender at cycles 1, 4 and 7 of the study, and within gender, over time, could not be reached but partial scalar invariance (L5) was attained at the cost of relaxing the constraint of equal item thresholds for several items. The five items (A, B, D, E and F) whose factors loadings were invariant across gender at cycle 1 also had invariant items thresholds at that cycle. However, whereas all items were metric invariant at cycles 4 and 7, only three items were also scalar invariant at cycle 4 (items C, E and F) or cycle 7 (A, B and E). Furthermore, in men, of the three items (A, D and E) that had similar factor loadings (respectively, .78, .92 and .87) over the 12 years of the study, two (D and E) also showed similar items thresholds (respectively, 1.24 and 1.79) whereas, in women, only one item (E) out of the four (A, C, D and E) that reached metric invariance over the study period also reached scalar invariance. It is noteworthy that, in men, the bad performance of the K6 items regarding longitudinal scalar invariance is mostly attributable to data from cycle 1 since five items (A, B, C, D and E) had equivalent items thresholds at cycles 4 and 7 whereas, in women, all items (except E) were non invariant between those cycles. In short, achievement of partial scalar invariance over a 12-year period is based on only two items (D and E) for men and one item (E) for women. No additional item had to be unconstrained at the scale factor level to reach partial invariance of the scale factor (L6). Thus, the pattern of scale factor invariance was identical to the pattern of scalar invariance discussed above and the final measurement model was the model of maximal partial invariance of the scale factor (L6).

### Longitudinal stability across gender

The gender-invariance of the longitudinal stability (L7) of latent psychological distress and of the residuals of the K6 items was confirmed. The overall level of longitudinal stability was similar but small for items A (so sad nothing could cheer you up), D (hopeless), E (worthless) and F (everything is an effort) over a 6-year (r = 0.05) and 12-year (r = 0.04) periods whereas it was similar but higher for items B (nervous) and C (restless or fidgety) (r = 0.20 over 6 years) (Table 8). The longitudinal stability of the latent distress factor was relatively high over a 6-year (r = 0.51) and 12-year (r = 0.52) periods.

Complete invariance of the latent factor variances (L8) was established across gender and over 12 years. However, complete invariance of the latent means (L9) could not be established. Longitudinal invariance of the latent means (L10) was established separately for men and for women but the latent mean was systematically higher for women than for men in each cycle. This implies that the gender difference in the latent factor means of distress stays the same, in standardized units, over the course of the study.

## Discussion

This study has uncovered several facets of the construct validity of the K6 across gender in different age groups and over a twelve-year period. Overall, the configural and metric invariance of the K6 across gender at cycle 1 suggest that women and men use the same conceptual framework in their appraisal of psychological distress, as defined by the K6, and that the symptoms described by the items of this scale have a similar meaning in women and men. However, higher levels of measurement and structural invariance were reached only after the constraint of equivalence was relaxed for various parameters of some items of the K6. This partial invariance implies that women and men slightly differ in the way they express their distress over their life-course but that these differences do not have a major impact on the construct validity of psychological distress as assessed with the K6. Findings on the longitudinal invariance of the K6 are less conclusive since the constraint of equivalence across gender and over time had to be relaxed for most items to reach partial scalar invariance. In addition, the Tau equivalence of the K6 items was not demonstrated. This suggests that summing up the score of each item to obtain a total score of distress may not be optimal. Kessler and his colleagues [1] came to the same conclusion. They initially proposed the computation of a score of distress based on a weighted sum of items; the weights being the severity score of each item. However, given the high correlation (r = .95) between the scores of distress based on the unweighted vs. weighted sum of items' score, they now recommend the unweighted sum of items' score [61].

This study confirms the construct validity of the K6 across gender in different age groups despite minor variations in the way women and men endorse some symptoms. A closer look at each item will provide some insight into the pattern of measurement and structural invariance of the K6 across age groups and over time.

The symptom "so sad that nothing could cheer you up" (item A) was endorsed by less than 40% of the respondents but its factor loading was relatively high (.77) making it an important contributor to the latent score of distress. The thresholds for this item were slightly higher for men than for women in the pooled sample and in cycle 4 of the survey but not in any specific age group. This pattern of threshold may be interpreted as follows: overall, given a similar level of latent psychological distress, men are less likely than women to feel so sad that nothing could cheer them up. Item A is the only item showing higher thresholds for men; thus "sadness" may be viewed as a more feminine symptom of distress although the gender difference in thresholds was small in cross-sectional analyses and at cycle 4.

The symptom "nervous" (item B) was endorsed by roughly 60% of the respondents and its contribution to the latent score of distress was lower yet not negligible (factor loading.56). Although women were more likely to report feeling nervous some of the time or more, the thresholds for this item were invariant across gender except for a small difference at cycle 4. Thus nervousness may be seen as a common symptom of distress in adult women and men, whatever their age.

The symptom "restless or fidgety" (item C) was endorsed by roughly 50% of the respondents and its contribution to the latent score of distress was similar to that of nervousness. This item was peculiar in that its thresholds varied across gender in the pooled sample and in the younger and older groups (but not in middle-aged); these thresholds also varied over the course of the study. The pattern of these thresholds suggests that, given an equivalent level of latent psychological distress, men are more likely than women to report restlessness and, in longitudinal perspective, both gender are more likely to report this symptom at cycle 1 (and in cycle 7 to a lesser degree) than in the other cycles. In addition, the lower loading of this item for men in cycle 1 vs. cycles 4 and 7 indicate that the relevance of this symptom to the total score of distress may not be stable over time.

The symptom "hopeless" (item D) was endorsed by a small minority of respondents, roughly 15% but, together with the symptom "worthless" (item E) it was the most important contributor to the latent score of psychological distress. This item is unusual in that its thresholds were not equivalent across gender in seniors only and over time. Furthermore, the pattern of gender difference in this item' thresholds was complex: the first three thresholds were lower in men than in women whereas the last threshold was higher in men. A tentative interpretation would be that, given an equivalent level of latent psychological distress, men are more likely than women to report low levels of hopelessness whereas they are less likely than women to report the highest level of hopelessness; in other words, at low levels of distress, hopelessness is more easily reported by men than women but it becomes less easily reported by men than women at the highest level of distress.

The symptom "worthless" (item E) was endorsed by a small minority of respondents, roughly 10% and, together with the symptom "hopeless" (item D) it was the most important contributor to the latent score of psychological distress. It was the only item to reach complete measurement and structural invariance in cross-sectional and longitudinal analyses.

Finally, the symptom "everything is an effort" (item F) was endorsed by roughly 50% of the respondents and it was a non negligible contributor (factor loading .63) to the latent score of psychological distress. The specific feature of this item is that its thresholds varied across gender only in the younger age group and in cycle 7 of the study. The pattern of these thresholds implies that, given an equivalent level of latent psychological distress, young men are more likely than young women to report that everything is an effort.

Interesting patterns emerged from the comparison of various parameters across items. On the one hand, items D (hopeless) and E (worthless) have the highest loadings and thresholds whereas, on the other hand, items B (nervous), C (restless or fidgety) and F (everything is an effort) have the lowest. Item A (sadness) stands in between these two groups of items. Thus the symptoms of hopelessness and worthlessness seem more central to psychological distress and more sensitive to high levels of distress, which may make them more suited to differentiate high levels of distress from very high levels of distress (especially, in women in the case of hopelessness). Similarly, the symptoms of nervousness, restlessness and effort appear more peripheral to distress and more sensitive to low levels of distress, which may make them more suited to differentiate an absence of distress from low levels of distress. In addition, items B (nervous) and C (restless or fidgety) differ from the other items by the slightly higher longitudinal stability of their residuals. Indeed, the stability of the residuals of the other items is very small (i.e., r = .04 and .05 over 6 years); thus the aspects of these items that are not related to distress may reflect the influence of context-dependent or state-related factors. In comparison, the residuals of items B and C were shown to be correlated and to be moderately stable longitudinally (i.e., r = 0.20 over 6 years), which suggests that these items partially tap into a slightly more stable characteristic, akin to trait anxiety.

The fact that several items of the K6 do not reach scalar invariance in longitudinal analyses is somewhat puzzling. A closer look at the specific patterns of longitudinal scalar invariance provides some insight into this issue. Most items were not equivalent at the scalar level over the twelve years of the study when men and women were viewed together. However, this lack of scalar invariance was mostly attributable to data from cycle 1. Indeed, looking at intra-group variation over time, one notes that, in men, five of the six items (i.e., items A, B, C, D and E) had similar items threshold at cycles 4 and 7 and thus reached scalar invariance whereas, in women, differences in items thresholds between these cycles were trivial for most non-invariant items (e.g., item B: 0.29 vs. 0.32 for the 1^st ^threshold). Furthermore, items thresholds tended to be lower at cycle 1 than at cycles 4 and 7, which suggest that, given a similar level of latent psychological distress, respondents were more likely to endorse some symptoms at cycle 1 compared to cycles 4 and 7. Thus, the problem of the longitudinal non-invariance of the K6 over a twelve-year period seemed to emerge from differences in items threshold between the first and subsequent cycles. This phenomenon where data collected in the first cycle of a survey differ from those collected in the following cycles is not uncommon [59]. It may signal some sort of panel effect [62] or Hawthorne effect where respondents become familiar with the survey procedure following the first interview and modify their behavior or attitude accordingly. Finally, it is noticeable that the differences in items thresholds at cycle 4 compared to cycle 7 were specific to women. Most of these differences were small and may not be meaningful. In addition, the interval of six years between these cycles seems too short to produce a detectable change in the way women perceive and express their distress. Still, the change in women's attitude may have started to take place in cycles 2 or 3, which were not investigated and the non equivalent items thresholds may be an indication that the construct validity of psychological distress is more likely to change over time in women than in men.

This study has some limitations that must be kept in mind to fully appreciate its findings. First, conclusions regarding the construct validity of the concept of psychological distress are not applicable to scales other than the K6, unless they contain a similar set of items. Second, this study was based on data collected in the general adult population; findings may not be generalized to clinical or adolescent samples. Third, over the 12 years of the study, 51% of respondents dropped out of the survey. This loss to follow-up is relatively small for such a long period of time and it is noticeable that the factor loadings were similar in cross-sectional analyses carried out with baseline data and at cycle 1 of longitudinal analyses. Still, a selection bias affecting the longitudinal analysis cannot be completely discarded. Finally, longitudinal analyses were performed exclusively on data from cycles 1, 4 and 7 and the observed gender differences may have occurred between these cycles.

## Conclusions

The K6 bears witness to the construct validity of the concept of psychological distress across gender in adults despite differences in the endorsement of some symptoms of distress in women and men. For instance, sadness may be viewed as a slightly more feminine symptom of general distress in adults whereas hopelessness is a more masculine symptom of lower level of distress but severe hopelessness becomes a more feminine symptom at the highest level of distress. In addition, some items seem to have a different pattern of non invariance across gender in different age groups: restlessness appears to be equivalent in middle-aged women and men but not in the other age groups whereas feeling that everything is an effort is more likely to be reported by young men than young women with the same level of distress but in older adults. These gender differences could be an indication that some culture-related norms regarding the expression of distress apply differently to specific age groups. However, these differences were small, their detection was made possible by the use of highly sophisticated analyses, and they did not markedly affect the construct validity of the K6 across gender over the life-course.

The longitudinal invariance of the K6 across gender was demonstrated at the cost of relaxing the constraint of equivalence for several items. Two phenomena seem to affect the invariance of the K6 over time. The first may be related to a panel or Hawthorne effect, which results in a modification of the behavior or attitude of respondents following the first research interview. In this study, it translates into lower factor loadings and items thresholds for some items at cycle 1 than in cycles 4 and 7. The second phenomenon is associated with a gender difference in the evolution of the construct validity of psychological distress and of the K6 over time, more precisely in the non equivalence of the thresholds of some items in women between cycles 4 and 7. The combined effect of these two phenomena made the demonstration of the longitudinal invariance of the K6 more complex but did not jeopardize the construct validity of this scale.

In conclusion, findings from this study suggest that the K6 is a gender-neutral scale and that the higher mean level of psychological distress observed in women may reflect a true gender difference in distress. Further researches are needed to unravel the factors underlying this widespread gender difference. This issue should not be neglected since psychological distress has become a popular indicator of the mental health of the population and a frequent outcome measure in the evaluation of health interventions.

## Competing interests

The authors declare that they have no competing interests.

## Authors' contributions

AD planned the study, reviewed the literature, supervised data analyses and the interpretation of findings, and assumed leadership for the writing of the manuscript. DBP carried out the analyses and participated in the interpretation of findings. AD and DBP wrote the manuscript. AM, MP and RB provided advice on data analyses and AM, RB and SK participated in the interpretation of findings and commented the completed version of the manuscript. All authors read and approved the final manuscript.

## Pre-publication history

The pre-publication history for this paper can be accessed here:

http://www.biomedcentral.com/1471-2288/10/68/prepub
